# Unique genetic architecture of CSF and brain metabolites pinpoints the novel targets for the traits of human wellness

**DOI:** 10.21203/rs.3.rs-2923409/v1

**Published:** 2023-06-09

**Authors:** Ciyang Wang, Dan Western, Chengran Yang, Muhammad Ali, Lihua Wang, Priyanka Gorijala, Jigyasha Timsina, Agustín Ruiz, Pau Pastor, Maria Fernandez, Daniel Panyard, Corinne Engelman, Yuetiva Deming, Merce Boada, Amanda Cano, Pablo García-González, Neill Graff-Radford, Hiroshi Mori, Jae-Hong Lee, Richard Perrin, Yun Ju Sung, Dominantly Inherited Alzheimer Network (DIAN), Alzheimer’s Disease Neuroimaging Initiative (ADNI), Carlos Cruchaga

**Affiliations:** Washington University, School of Medicine; Department of Psychiatry, Washington University School of Medicine, St. Louis, MO, USA; Washington University in St. Louis; Washington University in St. Louis; Washington University School of Medicine; Washington University, School of Medicine; Washington University, School of Medicine; Fundació ACE; University Hospital Germans Trias i Pujol; Stanford University; University of Wisconsin-Madison; University of Wisconsin-Madison; Research Center and Memory Clinic of Fundació ACE, Institut Català de Neurociències Aplicades-UIC, Barcelona; Research Center and Memory Clinic, ACE Alzheimer Center Barcelona. Universitat Internacional de Catalunya, Spain; Fundació ACE; Mayo Clinic; Department of Clinical Neuroscience, Faculty of medicine; University of Ulsan; Washington University in St. Louis; Washington University in St. Louis; Washington University in St. Louis; Washington University in St. Louis; Washington University, School of Medicine

## Abstract

Brain metabolism perturbation can contribute to traits and diseases. We conducted the first large-scale CSF and brain genome-wide association studies, which identified 219 independent associations (59.8% novel) for 144 CSF metabolites and 36 independent associations (55.6% novel) for 34 brain metabolites. Most of the novel signals (97.7% and 70.0% in CSF and brain) were tissue specific. We also integrated MWAS-FUSION approaches with Mendelian Randomization and colocalization to identify causal metabolites for 27 brain and human wellness phenotypes and identified eight metabolites to be causal for eight traits (11 relationships). Low mannose level was causal to bipolar disorder and as dietary supplement it may provide therapeutic benefits. Low galactosylglycerol level was found causal to Parkinson’s Disease (PD). Our study expanded the knowledge of MQTL in central nervous system, provided insights into human wellness, and successfully demonstrates the utility of combined statistical approaches to inform interventions.

## Introduction

Many metabolite levels are known to be heritable with a median heritability of 19.7%^1,2^. Hundreds of blood and urine metabolites have been associated with a number of loci through metabolite genome-wide association studies (MGWAS)^1–12^. The two recent large blood MGWAS included 8,299 and 14,296 individuals and identified hundreds of metabolite-signal associations^2,13^. A large-scale German Chronic Kidney Disease (GCKD) urine study with 1,627 participants identified 240 associations at genes with enriched kidney and metabolism-relevant cell type expressions^9^. These studies not only identified causal metabolites for common diseases, including bone mineral density, asthma, and chronic kidney disease, but also illuminated the role of common variants at genes of inborn error of metabolism (IEM) in building similar phenotypic features observed in IEM patients^2,13^. These studies contributed to the understanding of common diseases and complex human traits and nominated causal metabolic reactions to the phenotypes. However, very few MGWAS have been performed using neurological-relevant tissues.

To this date, the genetic architecture of central nervous system (CNS) metabolite levels has never been characterized, except for an exploratory study in cerebral spinal fluid (CSF)^14^, which is a commonly used proxy for brain tissues. The CSF and brain metabolite concentrations might be distinct from blood due to multiple factors, such as blood-brain barrier (BBB) and the metabolism of brain cells^15^. The tissue-specific genetic effects on transcript and protein levels in brain and nonbrain tissues may also transfer to downstream pathways that affect metabolite levels^16,17^, leading to brain and CSF-specific genetic regulations of metabolites. While the CSF and blood level of many metabolites, such as some amino acid derivatives and xenobiotics^18,19^, are highly correlated, some metabolites like tryptophan and kynurenic acid^20^ showed low correlation and others even had inverse correlation such as gamma-glutamylglutamine^19^, suggesting that peripheral metabolism can influence CSF yet do not govern CSF metabolome. Understanding the genetic architecture of CNS metabolite levels can provide additional information not captured in blood or urine. A recent MGWAS using CSF identified metabolite perturbations implicated in neurological disorders^14^. Although promising, this study had a limited sample size (N=291), which resulted in a small number of findings. Larger scaled studies are needed to comprehensively understand the etiology of brain-related traits and disorders as well as to identify their causal metabolites and potential drug targets.

Here, we conducted MGWAS using large and well characterized CSF (N=2,602) and brain (N=1,016) datasets, to first determine the overall genetic architecture of metabolite levels in CSF and brain and to compare them with other tissues in order to identify tissue-specific associations. We subsequently integrated the knowledge from MGWAS with colocalization, functional summary-based imputation (FUSION) and Mendelian randomization (MR) approaches to identify causal and druggable metabolites for related traits such as Alzheimer’s disease (AD), Parkinson’s Disease (PD), schizophrenia, cognitive performance, and bipolar disorder. The findings from this study can be leveraged to study other diseases and traits, informing causal and druggable targets.

## Results

### Study design

For CSF, we performed MGWAS using a three-stage study design: discovery, replication, and meta-analyses. The discovery stage included 1,224 unrelated non-Hispanic white (NHW) samples from the Knight Alzheimer Disease Research Center (Knight-ADRC), Dominantly Inherited Alzheimer Network (DIAN) and the memory and disorder unit at the university hospital Mutua de Terrasa, Spain (Barcelona-1) cohorts (Supplementary Fig. 1). The replication stage included a total of 1,378 unrelated NHW samples from the Alzheimer’s Disease Neuroimaging Initiative (ADNI), Fundació ACE Alzheimer Center (ACE) and the Wisconsin CSF study cohorts (WADRC and WRAP)^14^ (detailed information of the cohorts can be found in Supplementary Table 1). Metabolomics data of all cohorts were generated using the Metabolon HD4 platform. After rigorous quality control (QC; see methods and Supplementary Fig. 2, Supplementary Table 2), a total of 440 metabolites passed QC (329 non-xenobiotics, 59 xenobiotics and 52 minimally characterized metabolites; minimally characterized metabolites include unknown metabolites and partially characterized metabolites). In order to compare the CSF genetic architecture of metabolites with that of brain, we also performed a large-scaled meta-analyses of brain MGWAS that included a total of 1,016 unrelated NHW participants from three cohorts (Knight-ADRC and DIAN, N = 405; ROSMAP, N = 415; MAYO, N = 196; Supplementary Fig. 1). A total of 962 metabolites (779 non-xenobiotics, 74 xenobiotics and 109 minimally characterized metabolites) were included in the analyses (Supplementary Fig. 2, Supplementary Table 3). Of these 962 metabolites, 360 passed QC in CSF and therefore 602 were uniquely analyzed in brain (Supplementary Fig. 3). Following the identification of associations, we performed deep characterization and functional annotation to identify the effector genes.

In this study, we not only compared the genetic architecture of CSF, brain, blood and urine, but also determined novel *vs*. known associations and loci by comparing our results with the largest CSF, plasma and urine MGWAS available at the moment of the study. Furthermore, the genetic regulators for brain and CSF metabolites were used to identify genetically dysregulated metabolites (via TWAS/Fusion) and to uncover metabolites causal for 12 neurological and 15 non-neurological traits or disorders through colocalization and MR (Supplementary Fig. 1, Supplementary Table 4). Each of these traits and disorders either has been linked to the central nervous system or is a risk factor for brain disorders.

### CSF and brain MGWAS identify hundreds of novel and tissue specific metabolite associations

**Figure 1:**
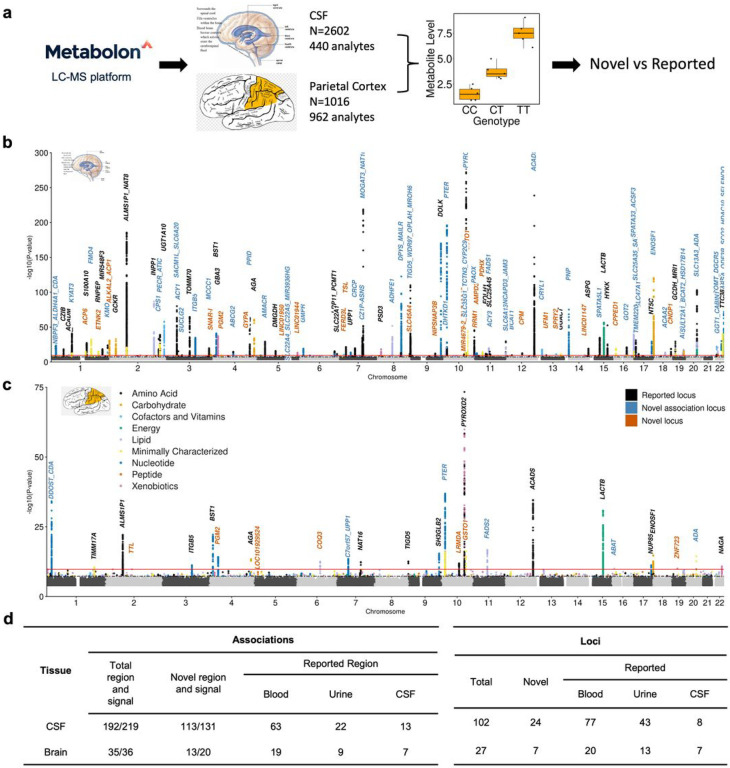
Mapping genetic regulators for metabolite level in CSF and brain identifies reported and novel association relationships and genetic loci. **a**, Schematic overview for MGWAS identification and replication. **b,** CSF combined Manhattan plot. The *x* axis denotes the chromosome and positions. The red line represents multiple test corrected significance threshold *P* = 2.79 × 10^−10^. **c,** Brain combined Manhattan plot. The *x* axis denotes the chromosome and positions. The red line represents multiple test corrected significance threshold *P* = 1.74 × 10^−10^. **d,** Replication using multiple tissues we identified the number of reported and novel associations and loci for both CSF and brain. Novel signal in replicated association region was identified if non-colocalized (PP.H4 <= 0.6). The association A/B indicates: A: association region B: independent association signal.

We performed the largest MGWAS to date in both CSF (N = 2,602) and brain (N = 1,016) tissues. In CSF, significant associations (metabolites-genetic locus pairs) were defined as those with: 1) nominal significance in discovery and replication; 2) effects in the same direction; and 3) study-wide significance in meta-analysis (P < 2.79×10^−10^; Supplementary Fig. 1; See material and methods for additional details). Among 440 CSF metabolites we identified a total of 192 associations for 144 metabolites at 102 distinct loci (Fig. 1a&b, Supplementary Fig. 4a&5a, Supplementary Tables 5&6). The effect sizes of the index variants between discovery and replication showed high correlation (r = 0.97; Supplementary Fig. 6), indicating high replicability and that both stages contributed to the association. Of these 192 associations, 173 associations belong to 130 well characterized metabolites (123 non-xenobiotics and 7 xenobiotics) and 19 associations belong to 14 minimally characterized metabolites (Supplementary Table 2&5).

Given the potentially complex nature of certain associations, we performed conditional analysis to identify independent associations within the identified regions. To be considered as an independent signal, we required that the variant passed the study-wide threshold (P < 2.79×10^−10^) in the conditional analyses. Of the 192 associations, 13 had two independent signals, and seven had three independent signals (Supplementary Fig. 7a). Therefore, in CSF we identified a total of 219 independent association signals for 144 metabolites at 102 loci. Of the seven association regions with three signals, two were novel (Supplementary Table 5). One of them was an association between methylmalonate (MMA) and the *ACSF3* region (16q24.3). Its primary signal included a missense variant (rs11547019 - p.Ala17Pro) for *malonyl-CoA synthetase family member 3*. While the two additional signals did not include any SNP that modifies protein sequence, but harbored known expression quantitative loci (eQTLs;P < 10^−4^) for *ACSF3*. In the other case, three independent signals were found at the *CNDP1* gene region which was associated with homocarnosine, a substrate of *CNDP1’s* encoded enzyme. While the primary signal (rs56042934) was intronic to *CNDP1* with unknown mechanism of action, the two other signals cause benign (rs73973908) and deleterious (the lead variant rs140836083) missense changes to the enzyme. In addition, there could be an underestimation in the number of independent signals, because the use of study-wide threshold can be too stringent. In fact, associations with higher significance are more likely to have complex regions based on student’s t test (student’s t = 3.24, P = 4 ×10^−3^; Supplementary Fig. 7b). Regardless, these results indicated that several independent signals may regulate metabolites levels in the same locus through multiple independent events (some signals change protein sequence or even protein function, and others alter protein level by modifying mRNA level)^17,21^.

As our study was enriched for Alzheimer’s diseases (AD) patients, our results may have been affected by participants’ health status. We therefore conducted sensitivity analyses by performing MGWAS including either only healthy (N = 883) or AD individuals (N = 769) based on the biomarker status (amyloid/tau/neurodegeneration (ATN) classification (see extended results)). The effect sizes of the index variants showed high correlation with that of analyses included either controls-only (r = 0.96; P < 2.2×10^−16^) or ADs-only (r = 0.97; P < 2.2×10^−16^) individuals, indicating that the disease status minimally affected our identified associations (Supplementary Fig. 8; extended results).

In brain MGWAS, only associations that had the same direction of effect for two or more three cohorts (for shared metabolites) and had study-wide significance in the meta-analyses were considered significant (Supplementary Fig. 1). The brain MGWAS (n = 1,016 individuals and 962 metabolites) identified 35 associations for 34 metabolites at 27 loci (Fig. 1a&c, Supplementary Fig. 4a&5a, Supplementary Fig. 9, Supplementary Tables 7&8, see extended results). Conditional analysis identified one additional independent signal for cytidine (Supplementary Fig. 7c). Therefore, we identified a total of 36 independent association signals for 34 metabolites at 27 loci. Of these associations, 16 were identified in both, CSF and brain (PP.H4.abf > 0.8, Supplementary Table 9). Many more associations were identified in CSF compared to brain that could be due to either CSF and brain having different genetic architectures or simply because CSF had higher statistical power due to sample size. To address this, we examined the study-wide significant associations (180 associations for 133 metabolites) of the 360 metabolites present in both tissues. The effect size for these 180 associations showed a high correlation between CSF and brain (r = 0.81, p < 2.2×10^−16^; Supplementary Fig. 10f; extended results), indicating that the genetic architecture of metabolites levels is similar between CSF and brain. Additionally, we applied Mashr^16^ approach to compare study-wide associations between CSF and brain in direction and magnitude. We found that 90% of associations had the same direction of effect and 57% of associations shared effects in both direction and magnitude.

We then performed additional analyses comparing the overall genetic architecture of metabolite levels across four different tissues: brain, CSF, blood and urine, using mashr (Supplementary Fig. 11). We used the latest blood and urine MGWAS available at the time of the study^9,12^. For this comparison, we focused on the 247 metabolites that were tested across all tissues and their genome-wide significant signals. All tissue pairs had over 80% of consistent direction of associations, with the highest percentage in CSF and blood (91%; Supplementary Fig. 11b, Supplementary Table. 10). When both direction consistency and magnitude similarity (within 2-fold) were considered, CSF and brain showed the highest (57%) overlap of associations followed by blood and urine (32%). Additionally, brain and urine had more direction specific associations (effect direction in one tissue being different from other tissues) than other tissues, indicating unique genetic regulation in brain metabolism and renal function (urine metabolite levels). These findings emphasized the need to analyze brain-related tissues in MGWAS in order to better understand neurological diseases.

Finally, we examined whether the CSF and brain MGWAS led to any novel signal by comparing to the large blood and urine studies and the CSF study of which was included in our meta-analysis^1,8,9,12,14^. Of the 219 independent association signals (192 associations) in CSF, 88 signals (70 association regions) had been previously reported in at least one of the five large-scale Metabolon-platform based studies (PP.H4.abf > 0.6; Fig. 1d; Supplementary Fig. 12). We found that 97.7% of the 131 novel association signals (at 113 novel regions and 9 reported regions) originated from previously examined metabolites in blood or urine, suggesting that our associations may be specific to CSF. The 131 novel signals corresponded to 24 novel loci and 49 previously reported loci that were associated with different metabolites (Fig. 1d; Supplementary Fig. 13).

For brain, 16 independent signals (16 associations) of the 36 signals (35 associations) have been reported (Fig. 1d) in previous studies (PP.H4.abf > 0.6; Fig. 1d, Supplementary Fig. 13). Therefore, we identified 20 novel association signals (at 13 novel association regions and six reported regions) for 18 metabolites, in which six signals (4 novel loci) were for metabolites not analyzed in any previous study. In addition, these 20 novel association signals correspond to seven novel loci and six reported loci associated with different metabolites than the one identified here (Fig. 1d; Supplementary Fig. 13, see extended results).

### Pleiotropic loci and polygenic metabolites

**Figure 2:**
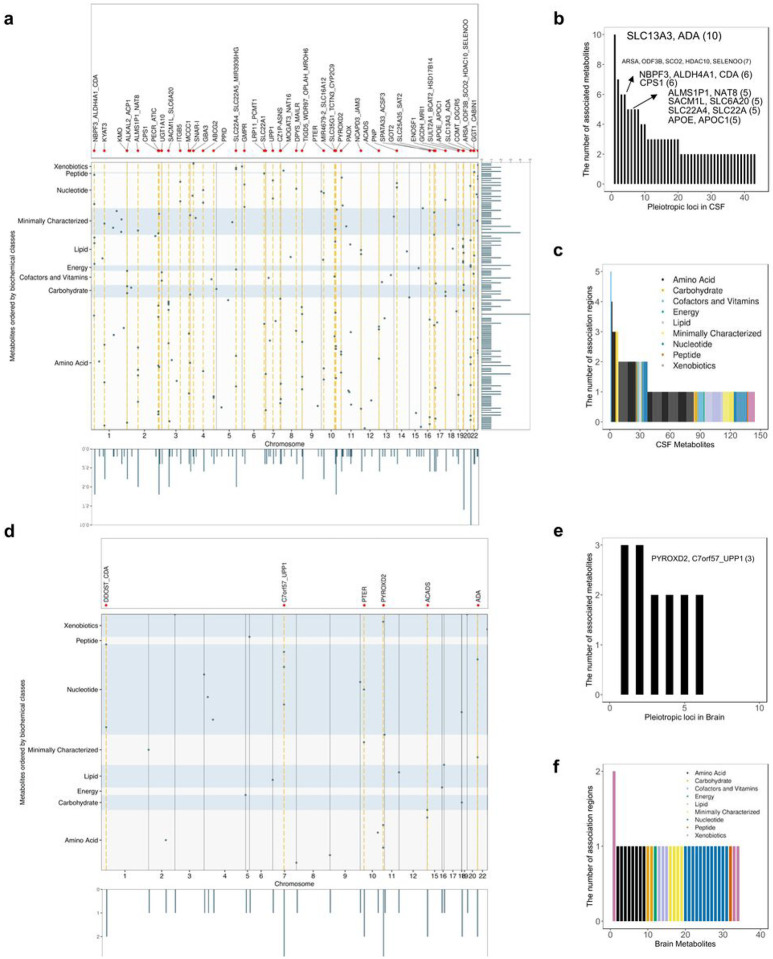
The characteristics of metabolite quantitative trait loci. **a(d),** Visualization of metabolites to loci associations in CSF and brain. The metabolites are ranked in y-axis based on their super pathways. Dots indicate the 192 association regions using their index variants. The pleiotropic regions were highlighted in orange. Pleiotropic regions were indicated by black vertical lines. **b(e),** Pleiotropy effect of CSF and brain metabolites quantitative trait loci. Each pleiotropic region (associated with more than one metabolite) was listed in the x axis. **c(f),** Metabolite polygenicity characterization in CSF and brain. Each metabolite was listed in the x axis.

Pleiotropic analyses can be instrumental to identify metabolites that are part of the same metabolic reaction or are unknown substrates, or products of one specific reaction. In CSF, 43 of the 102 identified loci were associated with more than one metabolite (Supplementary Table 6). Most of these loci were associated with two (23 loci) or three (10 loci) metabolites, although there were two loci with four metabolites, four loci with five metabolites, two loci with six, one locus associated with seven metabolites, and one locus associated with ten metabolites (Fig. 2a, b; Supplementary Table 6; Supplementary Fig. 14). The most pleiotropic CSF locus, located at *SLC13A3/ADA* gene region, was associated with ten metabolites (Fig. 2b, Supplementary Fig. 14a). This region was a complex region as there were two independent signals (based on r^2^ > 0.8): rs406383, intronic to *ADA*, was associated with N1-methyladenosine and rs439143, intronic to *SLC13A3*, was associated with nine different metabolites. Of these nine metabolites, seven were potential direct substrates of the transporter encoded by *SLC13A3*, being either amino acid derivatives or Krebs cycle components^22^, and the others were carnitine molecules secondary to Krebs cycle components.^22^

In brain, six of the 27 loci were pleiotropic and associated with either two (four loci) or three (two loci) metabolites (Fig. 2d–f). All six loci were identified as pleiotropic in CSF as well. The metabolites associated with these loci were often shared by both tissues, while additional metabolites were identified from brain due to either metabolites uniquely analyzed in brain or brain-tissue-specific associations not identified in CSF (see extended results).

The pleiotropic nature of many loci corresponds to known biological mechanisms, as in the case of *SLC13A3/ADA* locus, as in the case of *CPS1*, which encodes an enzyme catalyzing the first step of urea cycle. The variants in this region were associated with metabolites (Supplementary Fig. 14b, n = 6; i.e homoarginine, glycine, glutamine degradant, among others) that are part of urea cycle or alternative ammonia elimination pathways^23^. The *APOE/APOC1* locus was associated with five lipid metabolites, including cholesterol and four phosphatidylcholines (1,2-dipalmitoyl-GPC (16:0/16:0), 1-myristoyl-2-palmitoyl-GPC (14:0/16:0), 1-palmitoyl-2-stearoyl-GPC (16:0/18:0), 1-palmitoyl-2-palmitoleoyl-GPC (16:0/16:1)). Apolipoprotein E is known to interact with lipoproteins and function as cholesterol and phosphatidylcholines carrier^24^. *APOE* variants are one of the major genetic risk factors of Alzheimer’s disease (AD) and cholesterol has been associated with AD development downstream of Aβ and Tau pathology. Several studies also indicate that phosphatidylcholines may lower the risk for dementia and AD^25–27^. These five metabolites were also predicted to be associated with AD based on the MWAS analyses, and all of them were found lower in AD patients CSF based on differential abundance analysis (p < 0.05; Extended data Table 1, and extended results).

In addition, many metabolites were polygenic, meaning that multiple loci were associated with the same metabolite. In CSF, of the 144 metabolites with study-wide association(s), 37 metabolites were associated with multiple loci: 29 metabolites were associated with two loci, six metabolites with three, one metabolite methylsuccinoylcarnitine with four and one metabolite, bilirubin (E,E), was associated with five loci (Fig. 2c, Supplementary Table 5). The nominated effector genes (See section “*In silico* functional annotation of the CSF and brain associations”) for bilirubin (E,E) were *UGT1A6* (2q37.1), *GYPA* (4q31.21), *TWISTNB* (7p21.1), *FAS* (10q23.31), and *SPRY2* (13q31.1). *UGT1A6*, encodes an enzyme that transform bilirubin to water-soluble molecules and *GYPA* encodes the major intrinsic membrane protein of the erythrocyte, where bilirubin is generated^28^. Mutations in *FAS* leads to an autoimmune lymphoproliferative syndrome (ALPS) that is associated with hyperbilirubinemia^29^. However, the role of *SPRY2* in bilirubin metabolism is unknown but these findings suggest that it is also part of the pathways that produce or regulate bilirunin. The metabolite methylsuccinoylcarnitine was associated with four loci, which signals were predicted to affect *CPT2* (1p32.3), *SUCLG2* (3p14.1), *ACADS* (12q24.31), and *SLC13A3* (20q13.12) (Fig. 2c, Supplementary Table 5). Its association with *CPT2* has been reported previously, yet the mechanism is unknown. The other three loci were novel, and their nominated functional genes were implicated in the metabolism of methylsuccinoylcarnitine. Both *SUCLG2*, encoding a succinyl-CoA ligase, and the metabolite is involved in succinyl-CoA pathways. Mutation in *ACADS* causes short-chain acyl-CoA dehydrogenase deficiency and methylsuccinate level was altered in this disorder^30^. The *SLC13A3* encoded protein can transport succinate, which is a building block for methylsuccinoylcarnitine. This is the first time these genes have been linked to bilirubin and methylsuccinoylcarnitine levels. Additional functional analyses will be needed to characterize them in the context of these polygenic metabolites.

### In silico functional annotation of the CSF and brain associations

**Figure 3:**
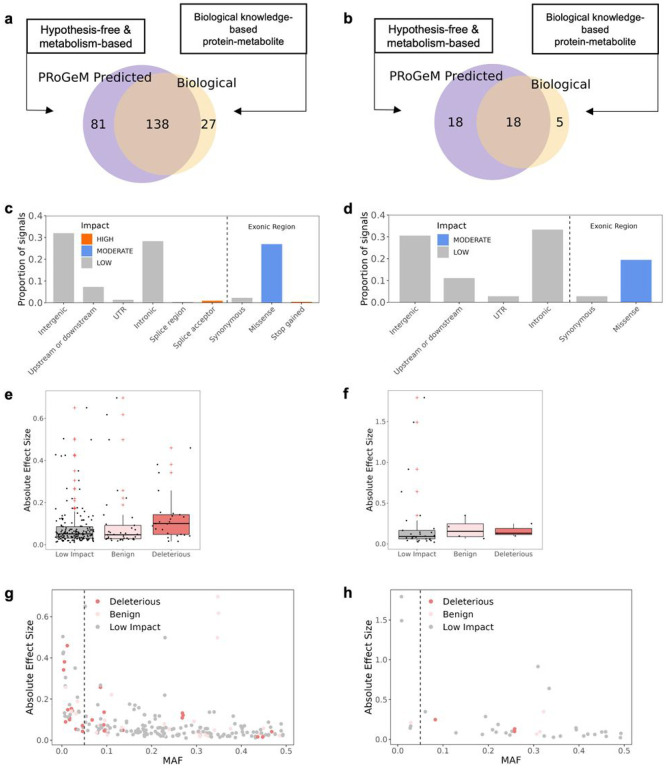
Functional characterization of genetic regions associated with CSF and brain metabolite levels. **a,** Identification of effector genes for the CSF MGWAS. **b,** Identification of effector genes of brain MGWAS. **c,** Distribution of functional annotations of CSF metabolite association signals. **d,** Distribution of functional annotations of brain metabolite association signals. **e,** Comparison of absolute effect size amongst impact level of the CSF association signals. **f,** Comparison of absolute effect size across impact levels of the brain association signals. **g,** Comparison of CSF signals’ effect size with minor allele frequency (MAF). **h,** Comparison of brain signals’ effect size with minor allele frequency (MAF).

To identify the effector gene for each association, we applied two complementary strategies (Fig. 3a, b). The first strategy is based on the ProGeM^31^ program which incorporates both genetic annotation and broad metabolism relevance; it prioritizes a gene if 1) the associated signal (the sentinel variants and its tagged variants (r^2^ > 0.8)) leads to a change in protein sequence, 2) the gene in the loci belongs to metabolic pathways, 3) the gene that harbors an eQTL overlaps with the association signal, and 4) it is the nearest gene to the sentinel variant (Supplementary Fig. 15)^31^. The second strategy is based on the manually curated biological knowledge, which relies on metabolite-gene relationship from KEGG^32^, GeneCards^33^, and HMDB^34^ databases.

For the 219 CSF signals, the ProGeM-strategy nominated 130 genes for 219 signals and the knowledge-based strategy nominated 89 genes for 165 signals (Fig. 3a). For brain, the ProGeM strategy nominated 29 genes for 36 signals and the knowledge-based strategy nominated 17 genes for 23 signals (Fig. 3b). Both strategies provided consistent predictions, with the same effector gene being nominated in 83.6% and 78.3% of CSF and brain associations (Fig. 3a, b). In case of discordance (27 CSF and 5 brain associations), the gene nominated from the biological knowledge-based strategy was prioritized over the ProGeM, as we confirmed that the ProGeM-strategy nominated gene was not biologically meaningful to the metabolite (see extended results).

Once the effector gene was nominated for each association signal, we categorized the associations firstly based on the location and consequence of variants to the effector gene, and subsequently based on eQTLs to the effector gene. Categorizing by consequence to the nominated genes, the association included a protein-sequence-altering variants (missense or splice acceptor variants) in 28.3% of CSF (62 association signals mapped to 39 genes) and 19.4% of brain associations (seven association signals mapped to 6 genes; Fig. 3c, d, Supplementary Fig. 16, Supplementary Table 5). Of these, 25 of the 62 CSF and three of the seven brain associations were deleterious to protein functions, predicted by SIFT and PolyPhen^35,36^ and of these, ten CSF and two brain deleterious associations are novel. Based on CSF, loss-of-function or deleterious variants had higher effect sizes (deleterious *vs*. benign missense: t = 3.3, p = 2 ×10^−3^; deleterious v.s. non-coding: t = 3.1, p = 6 ×10^−3^) and lower minor allele frequencies (deleterious vs. benign missense: t = −3.2, p = 2 ×10^−3^; deleterious vs. non-coding: t = −3.2, p = 3 ×10^−3^) than those that were non-coding or were predicted to be benign (Fig. 3e–h). We identified that 58.9% of the CSF (129 signals mapped to 81 genes) and 72.2% of the brain association signals (26 signals mapped to 19 genes) included an eQTL (Supplementary Table 5). In 15.1% of the CSF association signals (29 signals in 21 genes) and in 11.4% of the brain association signals, the same prioritized gene was supported by both altered protein sequence and eQTL variant evidence (Supplementary Table 5&7).

Among the nominated effector genes, 91.8% in CSF (87.9% of unique genes) and 77.8% in brain (75.0% of unique genes) encoded enzymes or transporters (Supplementary Fig. 17c, d). In addition, 42.9% and 34.4% of the total nominated effector genes for the CSF and brain association signals correspond to cis-proteins, defined as enzymes and transporter (production, degradation, transport) for a specific metabolite (Supplementary Fig. 17a, b).

Pathway analyses using KEGG database with the nominated effector genes found enrichment of many metabolism-related processes. The 127 unique nominated effector genes in CSF were enriched for branched chain amino acid degradation (map00280: P = 6.6×10^−12^), alanine, aspartate and glutamate metabolism (map00250: P = 7.5×10^−9^), and pyrimidine metabolism (map0024: P = 2.0×10^−8^; Supplementary Fig. 17e). The 29 unique genes in brain were enriched for pyrimidine metabolism (map0024: P = 1.3 × 10^−5^) and drug metabolism (map00983: P = 4.9 × 10^−5^; Supplementary Fig. 17f).

Then, we investigated if the nominated genes showed an enrichment for any specific brain cell type. The cell type specificity was determined for each gene based on gene expression (see material and methods)^37^. We found that the nominated effector genes for the CSF associations were enriched for astrocytes (log2FC = 1.66, p = 4.7×10^−5^, Supplementary Fig. 18), which were the key regulators of brain energy metabolism^38^.

### Insights into brain-related phenotypes using genetically regulated metabolites

Metabolism dysregulation, observed in many disorders, can be part of the causal pathway and potentially be good targets for intervention. The plasma MGWAS by Chen et al. identified 95 causal relationships for 12 phenotypes (five phenotypes included in this study) including O-sulfo-l-tyrosine for PD and the ratio of choline phosphate/choline for AD^2^. The recent urine MGWAS study identified 684 relationships between 110 metabolites and 68 phenotypes (no phenotype overlapped with this study) through colocalization analyses^9^. An earlier CSF MGWAS study^14^ identified 19 metabolites-trait pairs for multiple neurological and psychiatric disorders including attention deficit hyperactivity disorder (ADHD)-malate and schizophrenia-N-delta-acetylornithione^14^, through metabolome-wide association (MWAS) analyses.

Here, we integrate our CSF and brain MGWAS data to identify potential biomarkers and causal for 27 brain and wellness-related traits or disorders (Alzheimer’s disease, alcoholism, cognitive performance, among others; Supplementary Table 4), by integrating 1) MWAS (FUSION-approach^39^), 2) colocalization^40,41^ 3) MR^42^ and 4) drug repositioning^43^. Previous studies have used each of MR, colocalization or MWAS approaches. Here we integrated all three approaches along with drug repositioning to identify causal and druggable metabolites for complex traits for the first time^2,9,13^.

**Figure 4:**
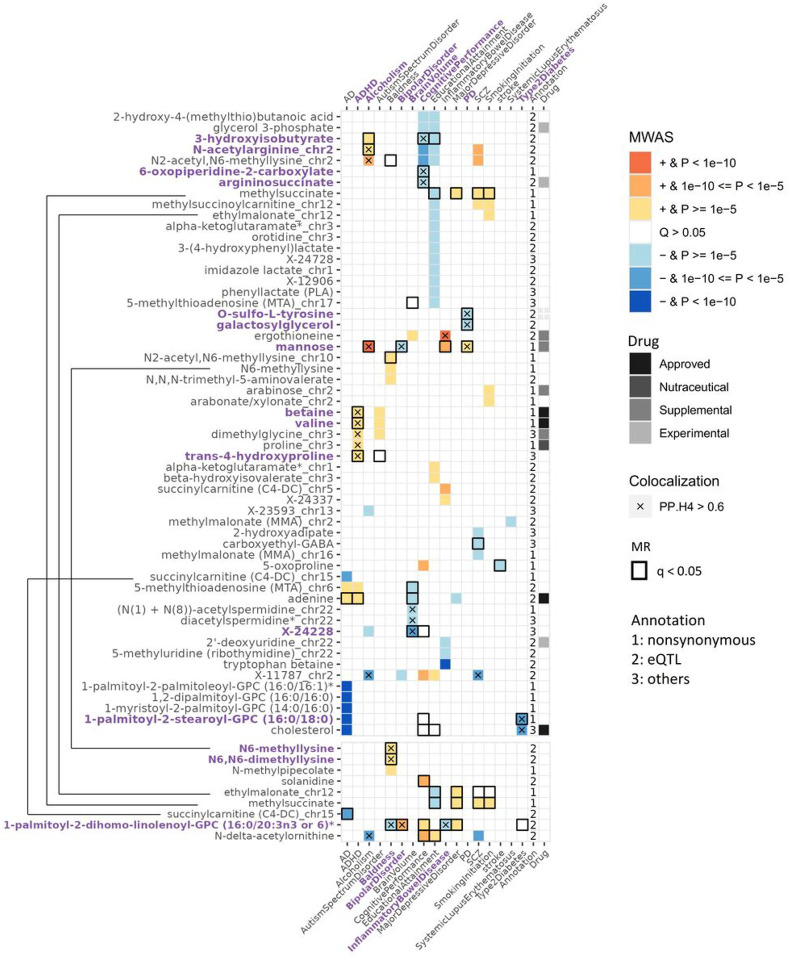
Associated or causal relationships between metabolites and complex traits/disease uncover insights into etiology. The plot showed associations between metabolite levels and traits identified from Metabolome-wide association study (MWAS) using CSF (top) and brain (bottom). Direction of effect and the strength of associations by P-value were represented by a range of colors. Colocalization were performed on each genetic locus through which the MWAS association were identified. Mendelian randomization (MR) tested all CSF metabolites with at least one association signal for all selected traits. The black bordered blocks illustrate significant findings from MR that passed FDR-correction. Metabolites, either monogenic (shown by metabolite name) or polygenic (shown by ‘metabolite_chromosome’ to specify the genetic region) were annotated by drug interests and effector gene categories.

To identify metabolites dysregulated with those traits, the FUSION approach was used to build metabolite level prediction models based on study-wide significant associations and performing association analysis between predicted metabolite levels and phenotypes. The weights for predicting metabolites were calculated for 92.4% (133/144) of CSF metabolites and 85.3% (29/34) of brain metabolites that had at least one heritable association region (Supplementary Table 11). Through this approach, we identified 62 CSF metabolite levels associated with 19 phenotypes including ADHD, alcoholism, bipolar disorder (128 metabolite-phenotype pairs), and nine brain metabolites associated with 12 phenotypes (22 metabolite-phenotype pairs; Fig. 4, Supplementary Figs. 19 & 20, and Supplementary Tables 12 & 13).

Both CSF and brain analyses identified seven metabolite-trait pairs, including four metabolites (succinylcarnitine (C4-DC), N6-methyllysine, methylsuccinate, ethylmalonate) and six traits (AD, baldness, educational attainment, major depressive disorder, schizophrenia, smoking initiation). Across tissues, these associations showed consistent effects in both direction and magnitude (Supplementary table 14). In total, we identified 140 unique metabolite-traits pairs in CSF and/or brain, in which only five were reported in the previous CSF MWAS study (Supplementary Table 15)^14^. Therefore, the remaining 135 metabolite-traits pairs are novel. We found the trait waist-to-hip ratio adjusted for BMI (WHRadjBMI) was associated with 21 metabolites (the largest number), education attainment with 19 metabolites, cognitive performance with 13 metabolites, schizophrenia with ten metabolites, and Alzheimer’s disease with nine metabolites.

To investigate whether the metabolite-phenotype associations identified through MWAS had the same functional variant for the metabolite and the phenotype, we performed colocalization analysis. Of the 128 CSF metabolites-trait (disease) associations, 26 pairs showed colocalization between the two traits (PP.H4 > 0.6; Fig. 4, Supplementary Table 16). Of the brain 22 associations, seven pairs showed colocalization (PP.H4 > 0.6; Fig. 4, Supplementary Table 17, see extended results).

To infer causal metabolites, we performed MR excluding highly pleiotropic regions (associated with > 5 metabolites)^44,45^. In CSF, we identified 38 metabolites causal for 22 traits after FDR correction (78 pairs; Supplementary Table 18). For brain, we identified 11 causal metabolites for 10 traits (20 pairs; Supplementary Table 19). In total, we identified 92 causal effects involving 46 metabolites and 22 phenotypes from both tissues. There were five causal relationships identified in both tissues, including for example succinylcarnitine for AD and HDL (Supplementary Table 20). In addition, we conducted a sensitivity analysis by performing MR using a more stringent method which removed all genetic regions associated with more than one metabolite. The sensitivity analysis identified 46 causal relationships between 20 metabolites and 18 phenotypes from both tissues (Supplementary Table 21–24, Supplementary Fig. 21).

The differences between the findings from the standard and stringent MR analysis come from how pleiotropic regions were defined. However, in some of these scenarios pleiotropic effects may identify relevant biological processes. For example, when a finding was pointing to an enzyme that catalyzes a specific metabolic reaction, changes in the activity of the enzyme will affect at least two metabolites: the direct substrate and the direct product. In some situations, it may affect more analytes if multiple substrates and products are involved. This could be the case where a signal (lead by rs17279437) affected *ACADS*^46^, which gene encodes an enzyme in beta-oxidation where fatty acids carried by carnitines were broken down to produce energy. This signal was associated with various metabolites involved in beta-oxidation pathways, like acylcarnitine related molecules, methylsuccinate^47^ and methylsuccinoylcarnitine, and fatty acids such as ethylmalonate. In the other scenarios, the signal may be driven by a metabolite channel or transporter, where genetic variants that decrease the activity of this transporter will lead to changes in levels of several metabolites. For example, the signal (lead by rs17279437) that affected a transporter encoding gene *SLC6A20*^48^ was associated with multiple substrates of the transporter, such as proline, betaine, and dimethylglycine. Therefore, although each single metabolite might not be causal, what is leading to the disease maybe the dysregulation of a specific metabolic process. However, these events will be identified as source of pleiotropic effects and were therefore removed from the MR analyses, leading to many false negative findings. Therefore, for MGWAS it may be necessary to reconsider how we may adjust the definition of pleiotropy by incorporating biological knowledge. To conclude, the intertwined nature of metabolic pathways often resulted in pleiotropic effects of signals, creating challenges for MR approaches, which may redirect us to identify metabolic reactions rather metabolites themselves.

In any case, we examined how many of the metabolites-traits pairs we found in our MWAS and MR were also reported in previous CSF, plasma or urine studies^2,14^. We replicated one of the three findings of the previous CSF study, which was brain ethylmalonate’s causal effect on schizophrenia, while the other two (N-delta-acetylornithine’s causal effect on cognitive performance and schizophrenia) were not replicated due to its pleiotropic signal at *NAT8*. Among 95 causal metabolite (or metabolite ratio)-phenotype relationships identified by the plasma Chen et al study^2^, we were able to analyze six pairs (three phenotypes and four metabolites), but were unable to replicate these findings: four due to tissue-specific findings, one due to study power difference, and one due to instrument variable selection difference (see extended results). At the same time, our analyses identified five causal metabolites-disease pairs that were previously tested but were not found as significant and therefore represent novel associations. These included bilirubin (Z,Z) for type 2 diabetes (T2D) and N-acetylhistidine for WHRadjBMI (Supplementary Table 18). These findings were driven by tissue specific MGWAS findings, highlighting the need to not just perform larger studies on plasma, but to expand these studies to additional tissues.

Then we integrated the findings from the three analyses: MWAS, colocalization and MR. In the CSF, 26 metabolite-trait pairs were significant for MWAS and MR, including nine pairs with colocalization evidence (Supplementary Table 25). These included six metabolites (Fig. 4) for seven traits (Alcoholism, bipolar disorder, WHRadjBMI, brain volume, cognitive performance, PD, and T2D). In brain, 11 pairs were significant in both MWAS and MR, in which two metabolites, N6-methyllysine and N6,N6-dimethyllysine showed colocalization with baldness (Supplementary Table 26).

For cognitive performance, we found causal associations with lower levels of two metabolites, 6-oxopiperidine-2-carboxylate and 3-hydroxyisobutyrate, based on all three analyses: MWAS, MR and colocalization (Fig. 4 & Supplementary Table 25). These associations were not found in the previous plasma studies because the genetic associations with these metabolites are CSF-specific. Some previous studies supported these metabolites influencing cognition. 6-oxopiperidine-2-carboxylate and 3-hydroxyisobutyrate have been linked to cognition in AD or epilepsy-specific studies^49,50^.

We also found that the higher levels of mannose may be causal to alcoholism, T2D, bipolar disorder, and PD based on MWAS, MR and colocalization analyses (Fig. 4 & Supplementary Table 25). The nominated effector gene for mannose, *GCKR*, was shown to affect both lipids and carbohydrates, including sphingolipids, glycerolipids, and serine (key connecter of amino acids to lipids, carbohydrates)^51^, suggesting that lipids and carbohydrates pathways may play an important role in alcoholism, T2D and PD. Mannose is known to be involved in alcoholism metabolism as it had an anti-steatosis role in alcoholic liver disease^52,53^. High mannose level at fasting have been associated with insulin resistance in diabetic individuals independent of obesity level^54^. In addition, *MBL2* (encodes mannose-binding lectin 2), which is implicated in mannose metabolism, has been found to be associated with bipolar disorder in genetic studies, supporting the causal role of this metabolite in bipolar disorder^55–57^. Our analyses indicate that lower mannose levels were associated with higher risk for bipolar disorder patients, and thus prescribing mannose, an available supplemental substance, may be useful to study as a potential intervention for bipolar disorder. Moreover, this is the first time that mannose was causally associated with PD based on literature.

Besides mannose, lower galactosylglycerol levels were potentially causal to Parkinson Disease (PD) through a signal that colocalized at *GALC* (Fig. 4 & Supplementary Table 25). The gene *GALC* encodes galactosylceramidase that removes galactose from ceramide derivatives, while the role of galactosylceramidase in galactosylglycerol metabolism in PD remains unknown. Knockout of *GALC* prevented alpha-synuclein accumulation in PD mice model, indicating that the enzyme galactosylceramidase may accelerate the development of PD by reducing galactosylglycerol^58^.

Additionally, lower xanthine level was predicted to have a causal effect for WHRadjBMI with support from MWAS, MR, and colocalization analyses (Fig. 4 & Supplementary Table 25). High level of xanthine oxidase activity, which reduced xanthine level, has been observed in obese individuals^59^. Therefore, xanthine as dietary supplement could be tested for potential intervention for individuals who suffer from obesity. Finally, an unknown metabolite X-24228 was found causal to brain volume, with their signals colocalized at a novel loci *CLDN16*. This gene plays a role in cell-adhesion, which is a crucial component in brain development^60^.

In brain, we identified N6-methyllysine and N6,N6-dimethyllysine to be causal for baldness, according to MWAS, MR, and colocalization analyses (Fig. 4 & Supplementary Table 26). The *PYROXD2* was the effector gene for both metabolites. Interestingly, CSF N6-methyllysine level neither shared the same causal signal with baldness nor had causal effect towards baldness, which could be explained by the tissue-specific association of N6-methyllysine (Supplementary Table 9).

Overall, we identified 11 high-confident metabolites-traits causal relationships (nine in CSF and two in brain) that were supported by the MWAS, MR and colocalization analyses. These associations were novel due to either the MGWAS signal being tissue-specific, or the metabolite not analyzed in other tissues. Previous studies only performed one or two of these three types of analyses to identify metabolites implicated in or causal to traits. Here, we reported metabolites-traits that were identified not only by these two approaches but also significant in the third method, which made our analyses more stringent. Many more metabolites-traits pairs were found in our analyses if we only require to be associated in two of those approaches: additional 43 metabolites-trait associations (34 in CSF and 14 in brain), were supported by two approaches (MWAS + Coloc; MWAS + MR), such as succinylcarnitine and adenine for AD, and (N(1) + N(8))-acetylspermidine and 5-methylthioadenosine (MTA) for brain volume and others (Extended results, Supplementary Table 27). In these 43 additional pairs, 41 were novel and therefore warrant future exploration.

### The Druggable metabolites

The metabolites identified in these analyses could be drug targets for improving disease outcomes or achieving desired phenotypes. Based on DrugBank database^43^,18 in 67 of the metabolites identified in the MWAS analyses were of pharmacological interest (Supplementary Table 28): there are six metabolites that are either approved drugs or being targeted by approved drugs. Betaine, which higher level was found to be associated with ADHD, Autism, and WHRadjBMI, is used for the treatment of homocystinuria to decrease elevated homocysteine blood levels^61^. Valine, which were also positively associated with ADHD, Autism and WHRadjBMI, is a crucial component of parenteral nutrition and the treatments such as Aminosyn II 7% was approved for premature infants^62^. Asparaginase treatments, such as pegaspargase, reduce asparagine levels and were approved for acute lymphoblastic leukemia. Lower asparagine level was associated with WHRadjBMI. Adenine (rejuvesol treatment), which higher level was found in AD, ADHD, and smaller brain volume, is approved for Sickle Cell Disease (SCD)^63^, suggesting that adenine may be implicated in multiple complex traits but with opposite effects. Therefore, if adenine is going to be targeted for therapeutic intervention, it will be important to track potential increased risk for other traits. Statins are FDA approved drugs to lower cholesterol levels in cardiovascular disease and others^64^. We found higher cholesterol level being associated with T2D and WHRadjBMI, and therefore the use of statins could also be used to treat T2D and obesity. In addition, five metabolites are commercial dietary supplements, and the three others are at experimental stages (See extended results for a detailed description of these findings; Supplementary Table 28). These results indicated that some of the metabolites identified as potential causal factors for these traits are druggable, but at the same time, due to the complex nature of the metabolism regulation, changing the levels of those metabolites may also increase risk of other diseases, and therefore a close monitoring of those potential secondary effects will be needed.

In addition, in some cases, the nominated effector gene, instead of the metabolite itself can be also druggable. For example, Tipiracil, an approved drug for gastric or colorectal malignancies, can inhibit the transferase activity of thymidine phosphorylase, which leads to higher levels of 2’-deoxyuridine. Our study showed that 2’-deoxyuridine was lower in inflammatory bowel disease (IBD) through *TYMP* locus (rs140522, p = 3.98 × 10^−57^) by MWAS, and consistently, a recent study showed that high uridine/2’-deoxyuridine ratio was causal for IBD^2^. Thus, the increase in 2’-deoxyuridine level by Tipiracil might provide therapeutic benefits in IBD, although experimental evidence would be needed to support this hypothesis. In another example, Belinostat and Panobinostat were pharmacologically approved inhibitors of Histone deacetylase 10, which regulates polyamine substrates including (N(1) + N(8))-acetylspermidine and diacetylspermidine (rs61748567, p = 5.56 × 10^−15^; rs143617749, p = 1.54 × 10^−33^). Higher levels of these metabolites were observed in brains with shrinked sizes based on MWAS. CNS injury was associated with an increase in N1-acetylspermidine level in rat brain, indicating a link between polyamine acetylation and impaired brain function^65^. Therefore, increased (N(1) + N(8))-acetylspermidine and diacetylspermidine levels by Belinostat or Panobinostat may likely lead to side effect of a reduced brain size.

## Discussion

We described a comprehensive, large-scale MGWAS study for 440 CSF metabolites in 2,602 individuals and 962 brain metabolites in 1,016 individuals, respectively. In CSF, we identified and replicated 219 independent signals (192 associations) for 144 metabolites in 102 loci, where 131 association signals and 24 loci were novel. In brain, we identified 36 independent signals (35 associations) for 34 metabolites, in which 20 signals were novel. Tissue specificity can be inferred from the observation that 59.8% of CSF independent association signals were novel yet only 2.3% of the novel signals were associated with newly analyzed metabolites. Our analyses indicate that CSF could be a good proxy to brain as we found a very high overlap across these tissues (correlation of effect size of study-wide association across tissues r = 0.82). In addition, the magnitude of the shared associations was more similar between CSF and brain, compared to blood or urine, indicating tissue-specific effects (Supplementary Fig. 11).

Most of the novel associations were driven either by metabolites measured only in CSF and brain or by genetic effects that were unique to these tissues that were not found in plasma and urine. Similarly, we observed a few metabolite-disease associations captured by plasma or urine studies, which were likely due to tissue-specific regulations. These observations have broader and more translational implications in understanding the biology of complex traits and identifying novel causal and druggable targets. It is instrumental to perform similar studies in more tissues besides analyzing larger studies in the same tissue or in different populations.

Following the discovery and replication of the genetic associations with CSF and brain metabolites levels, we pursued well-established and powerful statistical approaches to identify causal and druggable metabolites for various brain-relevant traits. These analyses also identified potential metabolism pathways involved in or causal to human phenotypes. For the 27 analyzed phenotypes, we identified 11 causal metabolite-to-trait effects for eight traits, including alcoholism, bipolar disorder, WHRadjBMI, cognitive performance, PD, T2D, and baldness. In addition, we identified 12 metabolites that are either druggable or already have compounds that could be repositioned. Of these, mannose was identified as a potential therapeutic target for bipolar disorder that requires further investigation, and the mannose dietary supplement may have opportunity for drug repositioning. Yet in other cases, metabolites were upregulated in diseased individuals or individuals with undesirable trait, which will redeem those compounds invalid in treating these disorders like AD, ADHD, autism, or improve traits like cognitive performance. In addition, drugs targeting the functional enzyme of a metabolite may provide therapeutic benefits. We found that Tipiracil may have potential to treat IBD by increasing 2’-deoxyuridine level, for which additional validation will be needed. Overall, we demonstrated the ability of MWAS approach to direct drug development.

There are some limitations of this study that warrant future efforts in the field. First, the study was focused on non-Hispanic white population, which may not discover ethnicity-specific findings from African, Asian or other populations. Second, despite the fact that we performed the first brain MGWAS analyses, the power of our brain study was relatively compromised by its limited sample size, and therefore created difficulty in identifying brain-tissue-specific associations and potentially interesting mechanisms of the brain.

In conclusion, our study described the largest CSF and brain MGWAS to date, identifying 219 associations signals for 144 CSF metabolites and 36 association signals for 34 brain metabolites. Through MWAS, MR, and colocalization, we found eight metabolites causal for eight brain related traits. Multiple of these metabolites are druggable or have compounds for drug repositioning. We filled in the knowledge gap in the lack of metabolome-wide genetic study in CNS and improved our understanding of the genetic risk loci for brain-related phenotypes in the metabolism perspective.

## Methods

### Study design and participants

The CSF MGWAS study included metabolomics and genotyped samples from 2,311 non-Hispanic white individuals, and a published MGWAS study^14^ using UW-Madison cohorts with 291 non-Hispanic white individuals. The CSF samples were collected from participants of five cohorts, including Memory & Aging Project (MAP) from Knight Alzheimer Disease Research Center (Knight ADRC), Dominantly Inherited Alzheimer Network (DIAN), Barcelona-1 (Longitudinal observational study from the memory and disorder unit at the university hospital Mutua de Terrasa, Terrassa, Barcelona, Spain), Alzheimer’s Disease Neuroimaging Initiative (ADNI), and ACE (ACE Alzheimer Center Barcelona, Barcelona, Spain; see extended results). The Knight-ADRC and the DIAN cohort belong to Washington University in St. Louis. The Knight-ADRC cohort included 47.18% of male, had an average age of 71.47 years, and had 70.22% of healthy controls (Supplementary Table 1). The DIAN cohort included 48.72% of male, had an average age of 38.39 years, and had 37.44% of healthy controls. The Barcelona-1 cohort included 53.52% of male, had an average age of 68.82 years, and had 1.88% of healthy controls. The ADNI cohort included 59.88% of male, had an average age of 73.69 years, and had 21.31% of healthy controls. The ACE cohort included 41.89% of male, had an average age of 72 years, and had 29.28% of healthy controls. The participants included in the UW-Madison cohort were 35.4% male, had an average age of 63.44 years, and were all healthy individuals.

The brain samples come from three different sources: the WU (Knight-ADRC and DIAN) cohort, which we generated genetics and metabolomics data and the two publicly available cohorts: Religious Orders Study/Memory and Aging Project (ROSMAP- syn25878459) and MAYO (syn26427298).

For CSF, the discovery stage included 1,224 samples from the Knight-ADRC, DIAN, and Barcelona-1. The replication stage included ADNI, ACE, and UW-Madison^14^. In the replication stage, ADNI and ACE were analyzed together using measured individual level data. The derived MGWAS summary statistics were combined with UW-Madison study by meta-analyses, which results were reported as the replication stage results. Lastly, we performed meta-analysis on discovery and replication to obtain the final results. These meta-analysis summary statistics were used for downstream analysis. Given that this is the first large-scale CSF MQTL study performed, the discovery-replication design helps to minimize the false positive discoveries.

In the Brain study, each cohort (WashU, ROSMAP and MAYO) were analyzed individually, followed by meta-analysis for shared metabolites between cohorts.

### CSF and brain samples

The 2,987 fasting CSF samples from 2,985 participants (from the five cohorts) were processed and stored at −80 °C. Samples were kept frozen until sent to Metabolon, lnc (Durham, NC). The Metabolon’s untargeted Precision Metabolomics^™^ LC-MS (liquid chromatography–mass spectrometry) analysis was used for metabolomics data generation. Metabolites values were first volume normalized and then median transformed to correct for each analytical method’s (“NEG”, “POLAR”, “POS EARLY”, “POS LATE”) day batches.

The 472 brain tissue samples (~50mg) were collected from the parietal lobe cortex of 459 WU participants, with 13 samples having technical replicates. All samples were shipped to Metabolon at the same time and measured in a single round of analysis. The metabolomics dataset was volume normalized, median transformed, and batch corrected as performed in the CSF. In addition, for the publicly available data, the 514 ROSMAP brain samples were collected from dorsolateral prefrontal cortex and 196 MAYO brain samples were collected from temporal cortex.

### Metabolite identification and quantification

All samples were measured using Metabolon’s untargeted Precision Metabolomics^™^ LC-MS (liquid chromatography–mass spectrometry). The technology is composed of four methods, including acidic positive ion conditions optimized for hydrophilic compounds, acidic positive ion conditions optimized for more hydrophobic compounds, basic negative ion optimized conditions, and negative ionization. All methods utilized a Waters ACQUITY ultra-performance liquid chromatography (UPLC) and a Thermo Scientific Q-Exactive high resolution/accurate mass spectrometer interfaced with a heated electrospray ionization (HESI-II) source and Orbitrap mass analyzer operated at 35,000 mass resolution. The scan range varied but covered 70–1000 m/z.

### Metabolomics quality control

The quality control of CSF dataset and three (WU, ROSMAP, MAYO) brain datasets followed the same pipeline, except for the slight differences in treating duplicated data. The duplicated samples in CSF came from longitudinal measures of the same individuals, and therefore were both kept for QC proposes but were removed in the MGWAS analyses. The duplicated samples in brain were technical replicates extracted at the same time. The brain replicated samples were merged through averaging the values, except for the case where only one sample has value. Given the difficulty in detecting metabolites in low abundance, the single value was kept only when it was close to the limit of detection (the lowest 10% of all values).

The initial steps of quality control assessed the missingness of each sample and each metabolite. A missing value can be due to sample and technical issues, metabolite not presenting in the sample, or the metabolite level being lower than detection limit. First, a sample with > 50% missingness was removed. Metabolites were defined by Metabolon to be either innate or foreign to human system as non-xenobiotics and xenobiotics. Non-xenobiotics are expected to be present in many samples, while xenobiotics can be largely missing due to their foreign nature. Therefore, only non-xenobiotics with > 80% missingness were excluded, while xenobiotics were not assessed at this step. Overall, taken non-xenobiotics and xenobiotics together, the average call rate for CSF metabolites was 89.6% and the average call rates for brain metabolites were 94.4% (WU), 93.9% (ROSMAP), and 97.0% % (MAYO; Supplementary Fig. 2).

In addition, due to the mixture of individual disease status (Control, AD, PD, FTD, aging) in all cohorts, the metabolites’ missingness could be caused by biological effect. Both fisher’s exact tests and linear regression were performed for each disease status group versus control group, and the consistently significant metabolites associated with disease status were recovered. For example, if fisher’s exact test identified higher missingness being associated with a disease and linear regression showed lower expression of a metabolite in diseased individuals, then the missingness could be caused by disease status instead of technical issue. None of the removed metabolites were recovered at this step because we found that their missingness were not caused by disease status. Given that CSF dataset consisted of five cohorts, the structure of each cohort was taken into consideration in several steps. The imputation of missing values was performed separately for each CSF and brain cohort. We performed imputation for non-xenobiotics using half-minimum value of the metabolite^66^, while xenobiotics were not imputed. Log10 transformation was applied to achieve approximate normal distribution. Moreover, given that metabolites with little variation throughout samples are non-informative for analysis, we removed metabolites that either have IQR equal to zero, or variance < 0.001. The outliers were determined separately by each cohort. Metabolite outlier values were defined as being outside the range of values from the first quantile minus 1.5-fold IQR to the third quantile plus 1.5-fold IQR. In addition, we removed metabolites with an overall limited number of values (N < 50) to ensure a sufficient power for analysis. Lastly, samples outliers, defined by > 5 std from the mean of principle component one or two, were excluded. Overall, taken non-xenobiotics and xenobiotics together, the average of call rate for CSF metabolites was 89.6% and the average call rates for brain metabolites were 94.4% (WU), 93.9% (ROSMAP), and 97.0% % (MAYO; Supplementary Fig. 2).

Given the discovery and replication design for CSF study, we further removed metabolites with < 50 values in each analysis stage to ensure the feasibility of each analysis. Moreover, the earlier CSF draw in longitudinal replicates were selected.

### Genotyping and imputation

Most CSF samples with Metabolon data had genotyped data: 2,727 in 2,985. In the brain study, 457 of the 459 WashU brain donors, 419 in 514 ROSMAP brain donors, and 196 in 196 MAYO brain donors had genotype data. The samples of Knight-ADRC, DIAN and ADNI were genotyped on multiple platforms from Illumina, including CoreEx, 660W, GSA, NeuroX2, OmniEx, Human610Quad, and BioBankUK. For participants genotyped multiple times, we selected one sample which platform that had the highest frequency of appearances in all samples. Samples of ACE cohort participants were genotyped solely using Axiom platform from Thermo-Fisher. Moreover, samples of ROSMAP and MAYO participants were genotyped using whole genome sequencing. Besides ROSMAP and MAYO cohorts, other cohorts’ genotype were imputed using the GRCh38 Version R2 reference panel on the TOPMed Server^67^. Before imputation, the direct genotyped variants were filtered based on the following criteria: (1) genotyping successful rate ≥ 98% per SNP or per individual; (2) MAF ≥ 0.01; and (3) Hardy–Weinberg equilibrium (HWE) (P ≥ 1 × 10^−6^). Then the DNA arrays results were lifted over to GRCh38, submitted to the server, which estimates haplotype phase using Eagle 2 software, and imputes based on the phased data. Imputed genotypes with imputation quality of *R*^2^ ≥ 0.3 were retained.

To select for non-Hispanic white and non-related participants, principal component analysis (PCA) was conducted using 1000 genome project ethnicity reference^68^, followed by pairwise genome-wide estimation of proportion identity by descent (IBD). Cryptic relatedness (PI_HAT >= 0.25) were identified, with one of the related pair samples being excluded. The participant filtering resulted in a selection of 2,311 CSF study participants, 405 WashU brain study participants, 415 ROSMAP brain study participants, and 196 MAYO brain study participants. Post-imputation quality control was performed for each study (CSF discovery study, CSF replication study, and three brain studies, respectively) based on the following criteria: (1) genotyping successful rate ≥ 95% per variant; (2) MAC >= 5; and (3) Hardy-Weinberg equilibrium (HWE) (p ≥1×10^−6^). The final cleaned imputed and genotyped data for each study were as follows: (1) CSF discovery: 10,495,409 variants in 1,224 participants; (2) CSF replication: 11,263,718 variants in 1,087 participants; (3) Brain WashU: 8,517,884 variants in 405 participants; (4) Brain ROSMAP: 10,888,777 variants in 415 participants; (5) Brain MAYO: 9,460,706 variants in 196 participants. Moreover, only the shared 10,307,658 variants between CSF discovery and replication studies were retained in the downstream analyses. In the brain study, for metabolites shared by two or three cohorts, only variants shared by at least two cohorts were retained for downstream analysis. For brain metabolites unique to a single cohort, all variants were retained.

### GWAS of CSF and brain metabolite levels

GWAS analysis was performed for each metabolite in each cohort using PLINK (v2.00a3LM)^69^. Linear regression of additive model was applied and controlled for multiple factors:

$$\text{CSF}\;\text{WashU}\;\text{discovery}:\text{log}10\left(\frac{\text{MetaboliteLevel}}{\text{Median}}\right)\sim \beta 0+\beta 1*\text{SNPdosage}+\beta 2*\text{age}.\text{at}.\text{CSF}.\text{draw}+\beta 3*\text{sex}+\sum_{j}^{4-13}\beta i*\text{geneticPC}+\sum_{j}^{14-21}\beta j*\text{cohort}.\text{genotypePlatform}+\varepsilon$$

$$\text{CSF}\;\text{WashU}\;\text{replication}:\text{log}10\left(\frac{\text{MetaboliteLevel}}{\text{Median}}\right)\sim \beta 0+\beta 1*\text{SNPdosage}+\beta 2*\text{age}.\text{at}.\text{CSF}.\text{draw}+\beta 3*\text{sex}+\sum_{i}^{4-13}\beta i*\text{geneticPC}+\sum_{j}^{14-18}\beta j*\text{cohort}.\text{genotypePlatform}+\varepsilon$$

$$\text{Brain}\;\text{WashU}:\text{log}10\left(\frac{\text{MetaboliteLevel}}{\text{Median}}\right)\sim \beta 0+\beta 1*\text{SNPdosage}+\beta 2*\text{age}.\text{at}.\text{death}+\beta 3*\text{sex}+\sum_{i}^{4-13}\beta i*\text{geneticPC}+\sum_{j}^{14-20}\beta j*\text{cohort}.\text{genotypePlatform}+\varepsilon$$

$$\text{Brain}\;\text{ROSMAP}\;\text{and}\;\text{MAYO}\;(\text{the}\;\text{same}\;\text{model}):\text{log}10\left(\frac{\text{MetaboliteLevel}}{\text{Median}}\right)\sim \beta 0+\beta 1*\text{SNPdosage}+\beta 2*\text{age}.\text{at}.\text{death}+\beta 3*\text{sex}+\sum_{i}^{4-13}\beta i*\text{geneticPC}+\varepsilon$$

All meta-analysis were performed using the inverse-variance-weighted (IVW) approach of METAL (2011-03-25 version, STDERR scheme)^70^. In the CSF study, internal replication study (ADNI and ACE) was first meta-analyzed with the previous CSF MQTL study^14^ using shared variants (MAF > 0.05 in the previous study), including only variants tested in the ADNI and ACE study^14^. Then the discovery- and replication-phase results were meta-analyzed. With one analyte removed due to inflation, a total of 440 metabolites’ meta-analysis GWAS results were reported. The standards of selecting significant signals were as follows: (1) Both discovery and replication phases P < 0.05. (2) Consistent in the direction of effects in two phases. (3) Meta-analysis result P < 2.79 × 10^−10^ (5 × 10^−8^ / 179 independent metabolites).

For brain, three cohorts were independently analyzed and meta-analyzed. The meta-analysis results were combined with independent analysis results for metabolites unique to a single cohort. With one analyte removed due to inflation, the brain results included a total of 962 metabolites. The criteria of selecting significant signals were as follows: (1) > 50% of cohorts captured the variant for shared metabolites. (2) Consistent direction of effects in cohorts for shared metabolites. (3) Meta-analysis (shared metabolites) or single cohort (unique metabolites) result P < 1.74 × 10^−10^ (5 × 10^−8^ / 287 independent metabolites).

### Identification and replication of associations between metabolites and genetic regions and metabolite loci

After filtering, significant variants for each metabolite went through forward selection to identify the index variants based on P-value ranking by extending 1Mb on each side of the index variant. Adjacent and overlapping regions of the same metabolite were merged and the region was extended beyond 2Mb. The ‘association’ or the equivalent term ‘association region’ was defined to be lead-index variant-to-metabolite relationship and its nearby region ( 1Mb on each side of lead-index variant). To identify genetic loci associated with the overall metabolite trait, unique lead variants for all metabolites were merged using the same method as for each metabolite. ‘Locus’ was defined as the region extending 1 Mb on each side of the lead-index variant that have 1 associated metabolites. These definitions were adopted from a previous study^10^.

To identify novel associations and locus, we curated several Metabolon platform-based large-scale studies, including Shin 2014, Long 2017, Schlosser 2020, Lotta 2021, Panyard 2021, and Yin 2022^1,8–10,12,14^. For each study, we extracted all significant association variants passed the multiple-test corrected P-value threshold for each specific study. To determine an association (region) as reported, we determine if the name-matched or Chemical ID-matched metabolite had at least one significant variant in the association region. A locus was determined to be reported if any significant variant in any metabolite fell inside the physical range of a locus. The non-replicated associations regions and loci were, as a result, novel findings. In addition, we tested if the identified association signals within replicated association regions were novel by performing colocalization analysis and we defined the novel signal to have a posterior probability under single causal variant assumption (PP.H4.abf) <= 0.6 with the association in other studies.

### Identify tissue specificity using mashr

Mashr^16^ was developed to compare significant associations amongst various tissue types in effect direction and effect magnitude. We compared metabolite-signal associations amongst four tissues, including CSF, brain, blood and urine. We first prepared the ‘random input’ (mimic all results) of association signals and then subset it to the ‘strong input’ (significant results) of associations. The ‘random input’ can be used as multiple test correction based on the protocol. Given that 91% of our identified associations nominated a metabolism gene as functional, we created ‘random input’ by extracting one variant per metabolism gene (ProGeM^31^ curated metabolism genes from GO, KEGG, MGI, orphaned, reactome databases) for each metabolite in the CSF study using cis-region of each gene (2Mb). Duplicated variants in each metabolite were removed, and variants within 2Mb distance were merged by selecting the most significant variant. For the analysis, we first identified shared metabolites in all tissues and then curated ‘random input’ for these metabolites. The ‘strong input’ was extracted from ‘random input’ by genome-wide significant threshold. The function ‘get_pairwise_sharing’ with default factor=0 was used to calculate the percentage of associations with the same direction shared by two tissues. The function ‘get_pairwise_sharing’ with factor=0.5 was used to calculate percentage of associations shared by two tissues not only in direction but also in magnitude. The default setting of factor=0.5 indicated that the two magnitudes were within two-fold of difference, which was defined by mashr to be similar. The effect sizes of an association in two tissues that met both the same direction and the magnitude ratio<0.5 were considered to be the same association.

### Variant annotation and effector gene nomination

The lead-index variant of each association together with its proxy variants (r^2^ > 0.8) were annotated using Variant Effect Predictor (VEP)^71^. The consequence of all variants from each signal was extracted from the VEP output. The consequences were prioritized based on impact level (high to low) and then based on the physical distance to the gene coding region (close to far). Then the consequences were grouped into the following categories: intergenic, upstream or downstream, 3’UTR and 5’ UTR, intronic, splice region, splice acceptor or donor, synonymous, missense, stop gained, stop lost. The results of VEP were input into prioritization of candidate causal Genes at Molecular QTLs (PRoGeM)^31^ to nominate effector gene(s). PRoGeM utilizes two strategies, “bottom-up” and “top-down”, followed by selecting the concurring gene(s) in two strategies as the candidate genes for an association. The “bottom-up” method prioritizes genes overlapping with the signal (between left-most and right-most proxy variant (+/− 5kb), the nearest genes (set to 10), if being protein-coding type, if its transcription being regulated by the variants based on GTEx eQTL (v7) database, and the variant impact based on consequences from VEP. The “top-down” method prioritizes genes within the distance range (set to 1Mb region to lead-index variant on both sides) by relatedness to metabolic pathways based on multiple databases. The steps of selecting candidate effector genes were as follows: 1) if the signal had a variant altering protein sequence or changing protein level dramatically (regulatory_region_ablation), which corresponded to high and moderate impact from VEP, then the respected gene is selected. If there were more than one gene, we chose genes overlapping with the signal’s LD region. 2) For other cases, prioritize genes selected by both bottom-up and top-down methods. 3) If no gene was shared by two methods, the nearest gene with an eQTL overlapping the metabolite association was selected. If no eQTL was found, the nearest gene was selected. 4) When co-occur genes were found, the nearest gene with an eQTL was selected. If no eQTL was found, the nearest gene was selected.

In addition to applying PRoGeM pipeline to predict the effector gene of metabolite-to-signal associations, we performed biological knowledge-based nomination of the effector gene for each association for named metabolites, from the 10 nearest protein-coding genes (if applicable) within a 2Mb window centered at the lead variant. If multiple genes were found relevant, we selected the nearer gene to the signal. We sourced from GeneCards^33^, HMDB^34^, the Uniprot database^72^, and the KEGG database^32^. For associations successfully nominated with a biological relevant gene, we defined the action of a gene to a metabolite to be cis-acting or trans-acting based on metabolism pathways. We defined cis-acting association to be either the gene encoding a direct transporter of the metabolite or the gene encoding an enzyme catalyzing a reaction that involved this metabolite. The trans-acting association can be the gene encoding a protein involved in the same pathway of the metabolite but didn’t directly catalyze the metabolite or the encoded protein being related to a metabolite based on literature with unknown mechanism.

Lastly, the biological knowledge nominated gene was prioritized over ProGeM nominated gene if discordance was found, and we confirmed that in each case, the ProGeM nominated gene was not biologically meaningful to the metabolite. When the biological relevant gene was missing, ProGeM nominated gene would be the effector gene. We further categorized effector genes based on its function based on enzyme and transporter. The information was obtained from the HMDB database together with GeneCards database.

The pathway analysis curating KEGG database was performed using MetaboAnalyst’s joint pathway analysis^73^ by inputting a list of effector genes.

### Cell-type enrichment analysis

Zhang et al published^37^ human brain RNA sequencing profiles for different cell types, including the mature astrocytes, neurons, oligodendrocytes, microglia/macrophages, and endothelial cells. The total expression was calculated by summing the average expression level for each gene in each cell types. For each gene, we divided the expression level in each cell type by the sum to get the proportion of gene expression in each cell type. We determine cell-type specific gene to be the gene which transcripts accounted for > 50% of all gene transcripts.

### Independent variant selection through conditional Analysis

Approximate conditional analysis was used to identify independent signals in each association, based on the LD structure of WashU participants’ genotype (CSF: 2,311 non-related non-Hispanic white individuals; Brain: 1016 non-related non-Hispanic white individuals from three brain cohorts). For each association, the GCTA^74^ COJO-slct function was applied to perform step-wide forward selection of approximate independent variants (P < 2.79×10^−10^ for CSF and P < 1.74×10^−10^ for brain; --cojo-collinear 0.9, and other settings using suggested values such as –cojo-wind 10000kb).

### Association with and causality to brain related wellness traits and diseases.

A total of 27 wellness traits or diseases were selected based on the relevance to brain and CSF that have large, well powered GWAS studies (Supplementary Table 10)^75^.

### Metabolome-wide association study (MWAS)

The analysis was conducted using FUSION^39^, which identifies association between a large GWAS phenotype and a functional phenotype that was measured in a limited number of individuals using the genetic components of both phenotypes. The metabolite phenotype (log10-median metabolite level) and covariates (age and sex) were input for weight calculation. We excluded the cohort and array covariates (which was included in the MGWAS) because a large number of covariates interfered with the MWAS and resulted in a large number of failed tests. Given the high significance of these associated genetic regions, the predicted metabolite levels would be reliable despite the slight bias from cohort and array differences. Weights of each metabolite association region (some metabolites had more than one weight) were calculated using all variants fell into the 2Mb region centered at the lead variant of the association. FUSION used GCTA-GREML^74^ program to calculate heritability of each association region. In CSF MGWAS, a total of 172 association region for 133 metabolites were heritable based on FUSION (default p < 0.01) using SNPID (chr:pos:ref:alt), and the weight for each association were calculated. Given that a number of traits and disease GWAS summary statistics were in rsID format, we additionally calculated weights based on variants with rsID available and calculated weights for 171 heritable association regions of 134 metabolites. The association tests were based on the genetic LD structure of CSF study 2311 non-related non-Hispanic white individuals. The association results between CSF metabolites and traits or diseases were controlled by FDR adjusted P-value < 0.05. Using brain MGWAS results, 29 association regions for 29 metabolites were heritable and had metabolite weights calculated for both SNPID and rsID form of variants. The association results between brain metabolites and traits or diseases were controlled by FDR adjusted P-value < 0.05.

### Colocalization

For each significant MWAS association between metabolite level and phenotype, colocalization analysis was performed for the region where metabolite association and phenotype risk locus overlap. The 2Mb window centered at the lead variant of trait/disease GWAS was selected. The variants of the region in trait/disease were used to curate variants in the MGWAS. If minor allele frequency (MAF) was available in trait/disease results, then the respective MAF were included for trait/disease and metabolite separately. If MAF was unavailable, then MAF from the MGWAS study was used for both. Coloc.abf function from the coloc R package version 5.1.0^41^ was applied using default priors with p_1_ = 1 × 10^−4^, p_2_ = 1 × 10^−4^, p_12_ = 1 × 10^−4^, p_1_ = 1 × 10^−5^. Probability for hypothesis four indicates the likelihood of the same causal variant shared by two phenotypes.

### Mendelian randomization

Two-sample mendelian randomization analysis (R package TwoSampleMR version 0.5.6)^42^ was conducted to estimate the causal effect of a metabolite (exposure) on trait or disease (outcome). In CSF, 144 metabolites with at least one association passed study-wide threshold were analyzed. In brain, 34 metabolites with at least one association passed study-wide threshold were analyzed. The instrument variables were derived from PLINK v1.90b6.4 clumping using respective CSF and Brain study participant genotype as LD reference. We used the default parameters for clumping (clump_kb = 10000, clump_r2 = 0.001) included in the R package. For metabolites with single instrument variable, Wald ratio method was applied. For metabolites with multiple instrument variables, inverse-variance-weighted method was applied. Both methods were the basic models in the R package. FDR correction was performed to correct for multiple metabolites and phenotypes tested. For phenotypes files that lack effect standard error information, we used “se.from.p” r package to infer standard error. In the main analysis, we excluded variants within *FADS* genes region and the highly pleiotropic regions associated with at least five^44,45^ CSF or brain metabolites identified from MGWAS. For the stringent analysis, we excluded variants within *FADS* gene region and all pleiotropic regions associated with at least two CSF or brain metabolites identified from MGWAS.

### Druggable targets

We identified related drugs for metabolites that was associated with at least one phenotype in the MWAS study. We identified metabolites that has been approved or tested as drug or metabolites that could be modulated through drug using DrugBank^43^. We also identified metabolites as dietary supplements that were commercially available. In addition, we identified the effector genes that overlaps drug targets using DrugBank^43^.

## Data Availability

Metabolomics and genomic data from the Knight ADRC participants are available at the NIAGADS and can be accessed at https://www.niagads.org/Knight ADRC-collection. Specific study ID for metabolomics data NIAGADS; ng00131. Data generated from the DIAN cohort can be requested at https://dian.wustl.edu/our-research/for-investigators/diantu-investigator-resources/dian-tu-biospecimen-request-form/; Metabolomics data from the ROSMAP participants are available at synapse.org and can be accessed at https://www.synapse.org/#!Synapse:syn25878459; Metabolomics data from the MAYO participants are available at synapse.org and can be accessed at https://www.synapse.org/#!Synapse:syn26427298.
